# Inflammatory Markers and Postoperative New-Onset Atrial Fibrillation: Prognostic Predictions of Neutrophil Percent to Albumin Ratio in Patients with CABG

**DOI:** 10.3390/diagnostics15060741

**Published:** 2025-03-16

**Authors:** Faruk Serhatlioglu, Yucel Yilmaz, Oguzhan Baran, Halis Yilmaz, Saban Kelesoglu

**Affiliations:** 1 Department of Cardiovascular Surgery, Faculty of Medicine, Nigde Omer Halisdemir University, Nigde 51240, Türkiye; faruks@erciyes.edu.tr; 2 Department of Cardiology, University of Health Sciences, Kayseri City Training and Research Hospital, Kayseri 38080, Türkiye; dryyilmaz@hotmail.com (Y.Y.); oguzhanbaran2009@hotmail.com (O.B.); 3 Department of Cardiovascular Surgery, Faculty of Medicine, Erciyes University, Kayseri 38030, Türkiye; halisy38@hotmail.com; 4 Department of Cardiology, School of Medicine, Erciyes University, Kayseri 38030, Türkiye

**Keywords:** postoperative new-onset atrial fibrillation, coronary artery bypass graft, neutrophil percentage to albumin ratio, inflammation

## Abstract

**Background/Objectives:** Postoperative new-onset atrial fibrillation (AF) (PNOAF) is the most common complication after coronary artery bypass graft (CABG), and its incidence has been reported as up to 50% in studies. In this study, we investigated whether there was a relationship between PNOAF and the neutrophil percentage to albumin ratio (NPAR) levels after on-pump CABG. **Methods:** A total of 454 patients who underwent CABG were included in the study. NPAR was calculated by dividing the neutrophil count by the albumin value. **Results:** It was determined that 93 patients developed PNOAF (20.4%). When the patient groups that developed and did not develop PNOAF were compared in terms of laboratory findings, C-reactive protein (CRP) values (4.0 mg/L (2.8–7.9) vs. 2.9 mg/L (1.1–6.7), <0.001), neutrophil/lymphocyte ratio (NLR) (2.2 (1.2–4.2) vs. 1.4 (0.7–3.1), <0.001), platelets-to-lymphocyte ratio (112 (72–177) vs. 92 (69–122), <0.001) and NPAR (2.29 (1.68–3.8) vs. 1.09 (0.79–1.81), <0.001), were found to be statistically significantly higher in the group that developed PNOAF. ROC analysis showed that the cut-off value for NPAR for the development of PNOAF was 1.86 with 78% sensitivity and 72% specificity (area under the ROC curve = 0.778, 95% CI (0.728–0.828), *p* < 0.001). **Conclusions:** NPAR, which can be detected by a simple venous blood test, has shown a strong predictive value for PNOAF in patients with CABG.

## 1. Introduction

Despite all the advances in invasive interventions on the coronary arteries and cardiac surgery over the years, coronary artery disease (CAD) remains a major cause of morbidity and mortality in developed countries and is increasing in other countries [1]. While optimal medical therapy is crucial to reduce symptoms, prevent the progression of atherosclerosis, and prevent atherothrombotic events, myocardial revascularization, including coronary artery bypass grafting (CABG), has a central role in the management of CAD. CABG can effectively relieve angina, eliminate myocardial ischemia and its adverse clinical manifestations, and reduce the risk of major acute cardiovascular events, including myocardial infarction and cardiovascular death [2]. Postoperative new-onset atrial fibrillation (PNOAF) is a common complication of CABG surgery with a reported incidence of 11–50% [3]. It usually occurs within 2–4 days after the procedure. PNOAF is associated with increased morbidity, mortality, prolonged hospitalization, and higher healthcare costs [4,5,6].

The major mechanisms underlying the occurrence of PNOAF are multifactorial, involving structural, electrical, and autonomic remodeling of the atria. Among these, the role of systemic inflammation is increasingly recognized as an important contributor to the pathogenesis of PNOAF. CABG triggers a systemic inflammatory response due to factors such as surgical trauma, myocardial ischemia-reperfusion injury, and extracorporeal circulation. These events lead to the release of pro-inflammatory cytokines such as interleukin-6 (IL-6), tumor necrosis factor-alpha (TNF-α), and interleukin-1 beta (IL-1β). In addition, tissue damage and the release of free oxygen radicals lead to increased oxidative stress and activation of neutrophils, platelets, and endothelial cells. All these factors have been shown to increase atrial fibrosis over time, promoting atrial electrical instability. This facilitates reentrant arrhythmias, one of the main mechanisms underlying PNOAF [7,8,9,10,11,12,13,14,15].

Identifying reliable predictors for PNOAF is crucial for implementing preventive strategies and improving patient outcomes. Therefore, several studies have recently been published focusing on the association of hematologic/biochemical indices with the occurrence of PNOAF. Systemic immune-inflammation index (SII), neutrophil/lymphocyte ratio (NLR), and C-reactive protein (CRP)-to-albumin ratio (CAR) are some of them, and in general, elevated markers have been reported to be associated with PNOAF [16,17,18]. However, a perfect marker has not yet been reached, and studies in this direction continue rapidly.

The neutrophil percentage to albumin ratio (NPAR), which was first described in 2016, was initially shown to be important in various patients with cancer, and there is increasing evidence for its role in the pathophysiology of cardiovascular diseases (CVD). However, there are no studies showing its association with PNOAF in the early post-CABG period. Our aim was to investigate whether there is an association between PNOAF and NPAR levels.

## 2. Material and Methods

### 2.1. Study Population

This study was planned as a single-center, retrospective, cross-sectional study. Patients were included in the study among patients who underwent coronary angiography (CAG) with a diagnosis of chronic coronary syndromes (CCS) who underwent CABG according to treatment guidelines and who had no previous history of arrhythmia [19]. (Must have 3-vessel CAD and/or left main vessel disease) Preoperative baseline demographics, comorbidities and laboratory parameters, operative data, and postoperative outcomes and complications were screened and recorded for analysis.

There were no restrictions on surgical technique (i.e., access route or graft) and preoperative/intraoperative and postoperative patient management as a result of participation in the study. Preoperative/postoperative medical/invasive care was performed according to current guidelines, and the medical treatment of patients who developed PNOAF was organized in light of current guidelines [20,21].

Exclusion criteria: age < 18 years, preoperative history of AF, atrial flutter or atrial tachycardia, left ventricular ejection function (LVEF) < 30%, atrial or ventricular complex arrhythmia (sinoatrial or atrioventricular blocks, AV nodal reentrant tachycardia, ventricular tachycardia, ventricular fibrillation), severe valvular disease (requiring valve repair or replacement), need for concomitant cardiac surgery (valve surgery, aortic surgery, surgical ablation, etc.), need for pre- or postoperative mechanical circulatory support (cardiopulmonary bypass machine, intraaortic balloon pump, etc.), need for reoperation or urgent CABG, hyperthyroidism, having urgent CABG, hyperthyroidism, having a heart defect, having a heart defect (valve surgery, aortic surgery, surgical ablation, etc.), pre- or postoperative mechanical circulatory support (cardiopulmonary bypass machine, intraaortic balloon pump, etc.), need for reoperation or urgent CABG, hyperthyroidism, having undergone surgery without pumps, New York Heart Association functional class III or IV heart failure, large left atrial (diameter > 55 mm), any inflammatory disease, and pericarditis.

This study was approved by the institutional ethics committee (2020/642). We conducted the study protocols in accordance with the ethical guidelines of the Helsinki Declaration.

### 2.2. Laboratory Analyses

Venous blood samples of all patients were collected from the antecubital region in the morning before CABG and on the day of hospitalization in the fasted state. Hematologic parameters (hemoglobin, platelets, and white blood cells (neutrophils and lymphocytes)) were counted using an automated hematology analyzer (Sysmex K-1000 Hematology Analyzer, Kobe, Japan). High-sensitivity C-reactive protein (CRP) tests and all routine biochemical tests were performed on another automatic analyzer (COBAS^®^ c701, Roche Diagnostics, Mannheim, Germany). NLR was calculated as the neutrophil count divided by the lymphocyte count, while the platelet-to-lymphocyte ratio (PLR) was calculated as the platelet count divided by the lymphocyte count. NPAR, a new inflammatory marker, was calculated as the ratio of neutrophil count to albumin.

### 2.3. Operative Technique

All patients were anesthetized with intravenous propofol, fentanyl, and rocuronium bromide according to a standard protocol after standard electrocardiography (ECG), monitoring arterial catheterization, and sPO_2_. Anesthesia management was performed using 60% oxygen and 6% desflurane inhalation. All patients underwent a standard median sternotomy using cardiopulmonary bypass (CPB) to access the aortic fat pad. CPB was achieved by cannulation (double-stage single cannula) of the ascending aorta and right atrium under moderate hemodilution (hematocrit 22–25%) and moderate systemic hypothermia (32 °C). Myocardial protection was achieved with topical hypothermia and anterograde cold blood cardioplegia (4 °C). The cardiopulmonary bypass flow rate was kept constant during the operations (2.1–2.4 L/min/m^2^, mean perfusion pressure 40–90 mmHg). When the aortopulmonary window was examined, a cross-clamp was placed on the aorta. The graft vessels were prepared for use by switching to cardiopulmonary bypass. An arterial graft was preferred, and the left internal mammary artery was used for revascularization of the left anterior descending artery. For the other vessels, anastomosis was performed with saphenous venous grafts from the legs. Systemic heparin (300 IU/kg) was administered during the operations. When necessary, heparin was supplemented to maintain an activated clotting time greater than 480 s and reversed with protamine at the end of the procedure. Blood transfusion was administered as needed (if hematocrit level < 20–25%).

### 2.4. Postoperative Management

At the end of CABG, hemodynamic and rhythm monitoring was performed in the intensive care unit (ICU). Patients were extubated with PaO_2_ >60 mmHg, FIO_2_ 40%, continuous positive airway pressure <5 mbar, PaCO_2_ <50 mmHg, and arterial pH >7.35 (mean 8 h). The choice of inotropic agents was determined by hemodynamic data. Serum electrolyte (magnesium, calcium, potassium) level imbalances were appropriately stabilized. Any hypocalcemia or hypopotassemia was immediately treated with extra therapy. Other routine postoperative medications for both groups were beta-blockers (metoprolol or carvedilol), nitroglycerin, pantoprazole, acetylsalicylic acid (300 mg/day), Clopidogrel (75 mg/day), N-acetyl cysteine, and nonsteroidal anti-inflammatory drugs. In the statin group, all extubated patients were started at the earliest postoperative period and continued as long as there were no contraindications. Diuretics, angiotensin-converting enzyme inhibitors, and warfarin were started gradually when clinically indicated. The need for perioperative blood products was determined on an individual, patient-by-patient basis; usually, blood transfusion was performed when hemoglobin was <9 g/dL [20].

### 2.5. Transthoracic Echocardiography

We performed transthoracic echocardiography on each participant both before and after CABG. All measurements were performed using a machine equipped with a 3.5 MHz transducer (Vivid 5, GE Medical Systems, Horten, Norway). We used two-dimensional echocardiographic measurements to assess LVEF and valvular pathologies. We assessed valvular pathologies with color Doppler in paraternal/apical two-chamber/four-chamber views. We used Simpson’s method to assess LVEF.

### 2.6. Definitions

Diabetes mellitus (DM): Postprandial blood glucose >200 mg/dL and/or fasting blood glucose > 126 mg/dL and/or use of anti-diabetic drugs.

Hypertension (HT): Systolic blood pressure/diastolic blood pressure at least 140 mmHg/90 mmHg or use of anti-hypertensive medication

Hyperlipidemia: Total cholesterol levels above 200 mg/dL and/or use of anti-hyperlipidemic drugs.

Smoker: Smokers who have smoked for at least 6 months/last year.

Atrial Fibrillation: Continuous ECG monitoring was provided to all patients in the intensive care unit during the first 48–72 h after surgery. All our patients underwent daily 12-lead electrocardiogram monitoring during their hospitalization. The diagnosis of PNOAF was based on any of the following: (I) Patient-reported symptoms suggestive of AF or detection of an irregular pulse during routine examinations, e.g., an ultrasound performed for other reasons; (II) Detection by bedside monitoring; (III) Detection based on ECG findings obtained for different clinical indications [21]. In case of suspected tachycardia, palpitations, or abnormal heart rhythm, a second 12-lead ECG was obtained and analyzed. The diagnosis of PNOAF was made by the presence of irregular RR intervals and the absence of P waves on the ECG.

## 3. Statistical Analysis

Data analysis was performed using TurcosaAnalytics v1.0.0 software (Melikgazi, Kayseri, Turkey) and SPSS (Version 24 SPSS Inc., Chicago, IL, USA). Data were evaluated for normal distribution using the Shapiro-Wilk test and histogram Q-Q plots. When comparing the two groups with and without PNOAF, an independent sample *t*-test was used for normally distributed parameters, and a Mann–Whitney U test was used for non-normally distributed parameters. Pearson χ^2^ test was used for categorical variables. ROC curve analysis was used to determine the sensitivity and specificity of NLR and NPAR values in the PNOAF group. PNOAF after CABG was calculated with univariate analysis. For multivariate regression analysis, parameters with a *p* < 0.05 in univariate analysis were included in the model. Regression analysis was done independently for each variable using inflammatory parameters to avoid multicollinearity.

## 4. Results

The clinical and demographic parameters of the patients at baseline are detailed in Table 1. A total of 454 patients, 297 (65.4%) of whom were male, were included in the study. Patients were followed up in the intensive care unit for a mean of 56 h. They were intubated for approximately 8 h on average and received inotropic support for an average of 23 h (the dose of inotropic medication was adjusted according to the patient’s needs). Among all participants, 93 developed PNOAF (20.4%). We found that 87 (93.5%) of these patients developed PNOAF during intensive care follow-up (within the first 72 h). Patients who developed PNOAF had significantly more history of DM and HT and significantly lower LVEF (*p* < 0.001, *p* = 0.008, and *p* < 0.001, respectively). Other baseline demographic characteristics, echocardiographic parameters, preoperative medications, and pre/postoperative variables did not show statistically significant differences between the groups (*p* < 0.05).

When the patient groups with and without PNOAF were compared in terms of laboratory findings, blood glucose values (109 mg/dL (86–153) vs. 88 mg/dL (78–106), <0.001), CRP values (4.0 mg/L (2.8–7.9) vs. 2.9 mg/L (1.1–6.7), <0.001), NLR (2.2 (1.2–4.2) vs. 1.4 (0.7–3.1), <0.001), and PLR (112 (72–177) vs. 92 (69–122), <0.001) were statistically significantly higher. We also found that the NPAR value, which is the subject of our study, was significantly higher in the patient group who developed PNOAF (2.29 (1.68–3.8) vs. 1.09 (0.79–1.81), <0.001). There was no statistically significant difference between the two groups in terms of other laboratory findings (*p* < 0.05) (Table 2).

In addition, we evaluated the role of various risk factors in the development of PNOAF using a multivariate analysis. We found that DM, HT, NLR, PLR, and NPAR were associated with the development of PNOAF in univariate analysis, and we performed multivariate logistic regression analysis with these variables (Table 3).

Multivariable logistic regression analysis showed that high NPAR levels (odds ratio [OR]: 1.017, 95% confidence interval [CI]: 1.009–1.025; *p* < 0.001), NLR (OR: 1.087, 95% CI: 1.027–1.256; *p* < 0.001), DM (OR: 1.913, 95% CI: 1.265–2.891; *p* = 0.002) and HT (OR: 1.470, 95% CI: 1.009–2.141; *p* = 0.045) were independent predictors of the development of NOAF.

**Figure 1:**
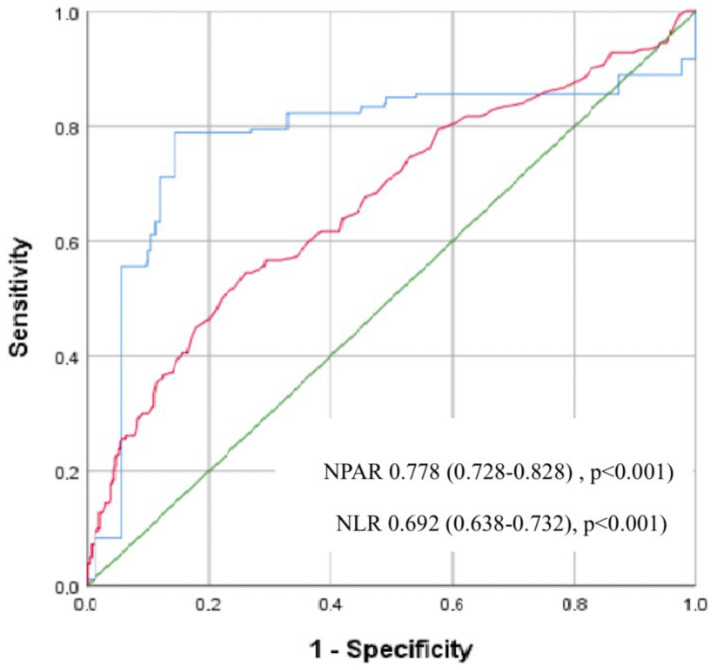
Receiver operating characteristic (ROC) curves for the neutrophil percentage to albumin ratio (NPAR) and the neutrophil to lymphocyte ratio (NLR) for predicting postoperative new-onset atrial fibrillation (PNOAF). (Blue line; NPAR; Red line; NLR; Green line; Diagonal line).

ROC analysis showed that for NPAR, the cut off value for PNOAF development was 1.86 with a sensitivity of 78% and a specificity of 72% (area under ROC curve = 0.778, 95% CI (0.728–0.828), *p* < 0.001), the cut off value for NLR to indicate the development of NOAF was 2.01 with a sensitivity of 72% and specificity of 69% (area under ROC curve = 0.692, 95% CI (0.638–0.732), *p* < 0.001) (Figure 1).

## 5. Discussion

This study evaluated the potential role of NPAR in predicting the development of early PNOAF in patients with CCS undergoing CABG. As a result of the study, NPAR emerged as a potential predictor of early PNOAF, and high NPAR values increased the risk of PNOAF development. This association emphasizes the importance of systemic inflammatory status in the occurrence of PNOAF.

When all cardiac surgeries are considered, AF is the most common arrhythmia in the postoperative period and is many times more common than in populations with similar demographic characteristics [22]. The development of PNOAF is associated with longer ICU stays, morbidity, and higher treatment costs [23]. Since the arrhythmia-predisposing (re-entrant mechanisms) environment will not persist in normal atria, the substrates resulting from pre/during/post-operative remodeling processes cause PNOAF through two main arrhythmogenic mechanisms: ectopic firing due to triggered activity and re-entry [24,25,26,27]. In addition, increased adrenergic activation, use of inotropic drugs, pericarditis, electrolyte disturbances, and some infectious/inflammatory conditions contribute to the increased risk of PNOAF [4,27,28,29,30]. In addition, age, left ventricular diastolic and systolic dysfunction, hypertension, and left atrial (LA) enlargement are considered important predisposing factors for PNOAF by causing atrial remodeling [31,32,33,34,35,36,37].

Although there are many speculations about the mechanisms leading to the development of PNOAF, some studies suggest that inflammation and increased inflammatory response play an important role in the development and progression of PNOAF [38,39,40]. Gibson et al. [16] and Ji et al. [41] showed an association between NLR and CRP levels in the development of PNOAF in patients undergoing CABG. Gungor et al. [42] proved that patients with high preoperative PLR levels had a higher risk of PNOAF. Yilmaz et al. [18] found a significant association between SII and PNOAF development. Aksoy et al. [17] reported that increased CAR values are associated with increased risk of PNOAF. In our study, we evaluated the possible associations between inflammatory markers such as NLR, PLR, CRP, NPAR, and PNOAF. Consistent with the literature, we found significant elevations in NLR, PLR, and CRP compared to those who did not develop PNOAF. Along with NLR, we also found a significant association in NPAR, which was the subject of our study, in patients with PNOAF.

Among inflammatory markers, NLR is among the first to be evaluated in patients with CAD and other diseases and is perhaps the most investigated marker. Gibson et al. [16] claimed that NLR can be used as a risk marker for AF development after CABG. Canpolat et al. [43] demonstrated it as a predictor for early recurrence of AF after radiofrequency catheter ablation. Karavelioglu et al. [44] showed a relationship between NLR and AF recurrence after medical cardioversion. Neutrophils represent nonspecific inflammation and are responsible for both initiation and progression [45]. They act directly on a cellular level as well as through the biomolecules they secrete and are involved in many steps of inflammation. Lymphopenia is a marker of poor nutrition and stress [45]. The NLR reflects this intercellular balance and can show the immune pathways as a measure of both systems.

Although NPAR is a much newer marker, it is increasingly being used in studies of patients with CVD. Sun et al. [46] reported an association between NPAR and one-year mortality in patients with CAD hospitalized in the ICU. Chen et al. [47] found an association between acute ischemic stroke severity and prognosis. Xu et al. [48] and Cai et al. [49] reported an association between NPAR and mortality in patients with AF. Zang et al. [50] found that elevated NPAR levels were an independent predictor of coronary slow flow phenomenon in patients with ischemia with no obstructive coronary arteries (INOCA). Cui et al. [51] found an independent association between NPAR and in-hospital mortality in patients with ST-elevation myocardial infarction (STEMI). Wang et al. [52] reported that NPAR was independently associated with mortality in congestive heart failure patients. Karaca et al. [53] claimed that NPAR is a newly identified promising inflammatory biomarker for predicting one-year major adverse cardiac and cerebrovascular events (MACCE) in non-ST elevation myocardial infarction (NSTEMI) patients undergoing revascularization therapy. Zhao et al. [54] showed that NPAR was independently associated with the severity of coronary atherosclerosis in chronic kidney disease patients. All these studies collectively emphasize the prognostic value of NPAR in various cardiac diseases. Elevated NPAR reflects increased inflammatory responses, and this is one of the critical factors affecting patient outcomes in CVD. In our study, we found that NPAR may be associated with the risk of developing PNOAF. This emphasizes the importance of assessing the preoperative inflammatory status in predicting postoperative complications.

The prognostic value of NPAR in the development of PNOAF may be attributed to the combined effects of neutrophils and albumin. As is well known, increased neutrophils represent the activation of inflammation, whereas albumin is a negative acute phase reactant. NPAR may represent a balance between both systems and may be considered as a measure of the response to stress and/or systemic inflammation. Another is the rapid movement of activated neutrophils through the bloodstream in response to the sterile tissue damage that occurs after CABG. They perform various tasks such as phagocytosis, degranulation (myeloperoxidase, etc.), respiratory burst, and formation of neutrophil extracellular traps (NETs) [55]. As a result, they can intensify tissue damage and inflammation by promoting the death of smooth muscle cells in the area [56]. Moreover, neutrophils are an early indicator of the proinflammatory cytokine milieu that causes hypercoaguloblasticity and can cause reperfusion injury [55].

Serum albumin is not only a marker of nutritional status but also an anti-inflammatory agent that scavenges free radicals. Albumin is also considered a negative acute phase reactant, and a decrease in albumin levels will increase blood viscosity and, therefore, increase oxidation of low-density lipoprotein cholesterol (LDL-C) and, hence, increase foam cell formation and inflammation [57]. The resulting mediators and growth factors exacerbate tissue damage. In conclusion, since albumin has anti-inflammatory, antioxidant, anticoagulant, and antiplatelet aggregation activity, low preoperative albumin levels may be associated with a high incidence of PNOAF, leading to decreased antioxidant defenses and increased vulnerability to oxidative stress [58].

## 6. Conclusions

NPAR, together with other composite inflammatory indices, such as NLR, PLR, and CRP, are valuable predictors of PNOAF. Incorporating inflammatory markers such as NPAR, a useful, simple, easily measurable, and inexpensive indicator of inflammatory status, into risk models may help identify patients at high risk for PNOAF and allow for targeted interventions to reduce the risk of PNOAF and improve postoperative outcomes. Future research should focus on improving these biomarkers and developing targeted interventions to reduce inflammation, thereby improving surgical outcomes for patients undergoing CABG and other cardiac procedures. However, larger multicenter studies are still needed to better analyze all possible determinants and the treatments that can be applied afterward.

## 7. Limitations

This study was planned retrospectively. Arrhythmia was monitored full-time only during hospitalization in the intensive care unit. Therefore, we may not have captured possible silent AF during ward follow-up. It was a single-center study with a relatively small number of patients. Although the operations were performed by a number of surgeons, the difference between operators cannot be excluded. Although the treatment algorithm was standardized for all subjects undergoing CABG and given to all patients as much as possible, we were not able to compare the specific role of patients’ medications, as different medications, different doses, and differences in duration of use were inevitable. Another limiting factor is that NPAR levels were only studied in blood samples obtained before CABG. Since it was not planned as a follow-up study, we did not evaluate follow-up periods and evaluated a single measurement. Finally, long-term follow-up data/blood measurements of patients were not included in the study.

## Figures and Tables

**Table 1:** Demographic characteristics of the study populations.

	CABG		
	AF+	AF−	*p* Value
Variables	(*n* = 93)	(*n* = 361)	
Age (years)	57 (49–64)	58 (42–67)	0.528
Men gender (*n*, %)	59 (63.4%)	238 (65.9%)	0.192
Diabetes Mellitus (*n*, %)	46 (49.5%)	131 (36.2%)	<0.001
Hypertension (*n*, %)	43 (46.2%)	123 (4.1%)	0.008
Dyslipidemia (*n*, %)	24 (35.8%)	141 (39.1%)	0.841
Smoking (*n*, %)	41 (44.1%)	178 (49.3%)	0.433
BMI (kg/m^2^)	26.9 ± 6.1	27.2 ± 3.1	0.952
Systolic blood pressure (mmHg)	132.54 ± 12.1	127 ± 6.45	0.347
Diastolic blood pressure (mmHg)	82.45 ± 0.86	77.53 ± 0.76	0.671
LVEF (%)	49.6 ± 12.3	52.45 ± 8.9	<0.001
Left atrium (mm)	3.9 ± 0.4	3.4 ± 0.6	0.02
Previous medications, *n* (%)			
Aspirin	61 (65.6%)	207 (57.4%)	0.373
Β-blocker	45 (48.4%)	183 (50.7%)	0.691
ACEI/ARB	34 (36.6%)	169 (46.8%)	0.552
Statin	32 (34.4%)	151 (41.8%)	0.431
Clopidogrel	12 (12.9%)	33 (9.1%)	0.116
Operative and postoperative parameters			
EuroSCORE II	4.35 ± 1.09	4.46 ± 0.7	0.097
By-Pass time (min)	116.5 ± 12.4	108.9 ± 9.6	0.791
Cross-Clamp time (min)	50.1 ± 6.5	47.3 ± 4.9	0.531
Number of bypass grafts	2.8 ± 1.7	2.5 ± 2	0.385
Duration of the hospitalization at the intensive care unit (days)	3.1 ± 0.8	2.98 ± 1	0.126
Extubation time (hours)	8.1 ± 4.1	8.9 ± 3.2	0.321
Intraoperative mortality (*n*, %)	-	-	
In-hospital mortality (*n*, %)	1	2	-

AF: atrial fibrillation, BMI: body mass index, LVEF: left ventricle ejection fraction, ACEI: angiotensin-converting enzyme inhibitor, ARB: angiotensin receptor blocker.

**Table 2:** Laboratory findings of the study populations.

	CABG		
	AF+	AF−	*p* Value
Number of Patients	(*n* = 93)	(*n* = 361)	
Glucose (mg/dL)	109 (86–153)	88 (78–106)	<0.001
Creatinine (mg/dL)	1.03 ± 0.2	0.92 ± 0.2	0.043
AST (U/L)	28.3 (20–32)	26.4 (19–29)	0.477
ALT (U/L)	20.1 (16–24)	20.7 (17–26)	0.684
Total cholesterol (mg/dL)	183.2 (152–216)	179.7 (154–211)	0.613
High-density lipoprotein cholesterol (mg/dL)	38.3 (29–46)	39.2 (31–45)	0.741
Low-density lipoprotein cholesterol (mg/dL)	126.8 (93–145)	116.5 (97–154)	0.328
Triglyceride (mg/dL)	131.7 (99–325)	136.1 (106–251)	0.082
Hemoglobin (mg/dL)	13.6 (11.9–14.8)	13.9 (11.3–15.1)	0.702
Platelets (10^3^/µL)	234 (194–292)	240 (190–302)	0.505
WBC (10^3^/µL)	7.5 (5.2–10.5)	7.1 (4.9–10.9)	0.394
Neutrophil (10^3^/µL)	7.4 (4.5–8.5)	4.0 (3–6.5)	<0.090
Lymphocyte (10^3^/µL)	1.9 (1.4–2.9)	2.6 (2.1–3.1)	<0.001
C-reactive protein (mg/L)	4.0 (2.8–7.9)	2.9 (1.1–6.7)	<0.001
Albumin (g/L)	3.6 (3.5–4.1)	3.9 (3.6–4.2)	0.055
Neutrophil-to-Lymphocyte ratio	2.2 (1.2–4.2)	1.4 (0.7–3.1)	<0.001
Platelets-to-Lymphocyte ratio	112 (72–177)	92 (69–122)	<0.001
NPAR	2.29 (1.68–3.8)	1.09 (0.79–1.81)	<0.001

CABG: Coronary artery bypass grafting, AF: Atrial fibrillation, AST: aspartate aminotransferase, ALT: alanine aminotransferase, WBC; White blood cell, NPAR; neutrophil percentage to albumin ratio.

**Table 3:** Univariate and multivariate logistic regression analyses of independent parameters for AF.

	Univariate Analysis			Multivariate Analysis		
	Odds Ratio	95% CI	*p* Value	Odds Ratio	95% CI	*p* Value
Diabetes Mellitus	2.080	1.414–3.060	<0.001	1.913	1.265–2.891	0.002
Hypertension	1.611	1.133–2.290	0.008	1.470	1.009–2.141	0.045
NPAR *	1.020	1.013–1.027	<0.001	1.017	1.009–1.025	<0.001
NLR *	1.122	1.051–1.199	0.001	1.087	1.027–1.256	<0.001
PLR	1.008	1.005–1.011	<0.001			
CRP	1.031	0.995–1.068	0.001			

NPAR: Neutrophil percent to albumin ratio, NLR: Neutrophil-to-lymphocyte ratio, PLR: Platelets-to-lymphocyte ratio, CRP: C-reactive protein. * These parameters were not entered into the model in order to prevent multicollinearity.

## Data Availability

The data that support the findings of this study are available from the corresponding author upon reasonable request.
